# Exploring how people achieve recommended levels of physical activity, despite self-reported economic difficulties: a sense of coherence perspective

**DOI:** 10.1186/s12875-024-02354-z

**Published:** 2024-04-04

**Authors:** Lisbeth M. Johansson, Eleonor I. Fransson, Hans Lingfors, Marie Golsäter

**Affiliations:** 1 Unit for Research and Development in Primary Care, Futurum - Academy for Health and Care, Region Jönköping County, Jönköping Academy for Improvement of Health and Welfare, School of Health and Welfare, Jönköping University, Box 1026, Jönköping, 551 11 Sweden; 2 School of Health and Welfare, Jönköping University, Box 1026, Jönköping, 551 11 Sweden; 3 Unit for Research and Development in Primary Care, Futurum - Academy for Health and Care, Region Jönköping County, Jönköping, Sweden; 4 Child Research Group, School of Health and Welfare, Jönköping University, Jönköping, Sweden; 5 Child Health Service and Futurum - Academy for Health and Care, Region Jönköping County, Jönköping, Sweden

**Keywords:** Sense of coherence, Physical activity, Deductive content analyse, Economic difficulties, Health promotion

## Abstract

**Background:**

The salutogenic theory forms the basis for health promotion and describes health as a continuum from a dis-ease pole of health to an ease pole. The core concept for the salutogenic theory is sense of coherence (SOC). For a strong SOC, general resistance resources, such as solid economic situation, are essential. The aim was to explore how people – despite self-reported economic difficulties – comprehend, manage and find it meaningful to achieve the level of physical activity recommended by World Health Organisation (WHO).

**Method:**

The study is based on interviews with people achieving the recommended physical activity (PA) level despite economic difficulties. The interviews were conducted at primary health care centres and family centres after a targeted health dialogue. We used a qualitative deductive content analysis based on sense of coherence as the main category, with the three generic categories of comprehensibility, manageability and meaningfulness.

**Result:**

The findings elucidate a pattern of a process. In this process, the participants comprehend their knowledge of the health benefits of PA and have a plan for performing their PA. They utilise their resources in order to manage to apply their knowledge and plan for PA in their lives despite their challenges. When PA becomes meaningful to them, they have an intrinsic motivation to perform it and experience its benefits.

**Conclusion:**

This study suggests a possible process that might help in achieving the recommended PA level among people with economic difficulties and other challenges. The findings might be used in health promotion work, such as targeted health dialogues in primary health care, to reduce health inequalities when supporting people who are not achieving the recommended levels of PA.

**Trial registration:**

Not applicable.

**Supplementary Information:**

The online version contains supplementary material available at 10.1186/s12875-024-02354-z.

## Background

The prevalence of non-communicable diseases (NCDs), such as cardiovascular diseases (CVDs), diabetes, cancer and chronic lung disease, is more common in groups with lower socioeconomic status (SES). These largely lifestyle-related diseases account for two-thirds of all premature mortality from NCDs in the European region, and affect the well-being of many people. It has been estimated that unhealthy lifestyle habits can explain at least 80% of CVDs and type II diabetes as well as 40% of all cancers, and it has been shown that a healthy lifestyle decreases the risk for all-cause mortality [1–6]. Moreover, physical activity (PA) can improve quality of life, mental health, memory function, vitality and mastery [2, 3, 7–9]. It is also associated with a reduction of the detrimental effects of stress [10]. For adults, the World Health Organisation (WHO) recommends a PA level of at least 150 to 300 min per week of aerobic PA of moderate intensity or at least 75 to 150 min per week of vigorous intensity, or a combination of these [2].

It has been observed that people from lower SES groups are more likely to spend less time in leisure time physical activity (LTPA) [11], and that high income is associated with a higher amount of LTPA [12]. Even having belonged to a low SES group during childhood is associated with less time spent in LTPA in adulthood [13]. Concerning engagement in PA, especially vigorous PA, the gap between people with low vs. high SES is widening [14]. Additionally, it has been observed that people with economic difficulties and a low PA also have lower self-rated health compared with those without economic difficulties [15].

Health promotion has been defined as a ‘process of enabling people to increase control over, and to improve, their health. To reach a state of complete physical, mental and social well-being, a person or group must be able to identify and to realise aspiration, to satisfy needs, and to change or cope with the environment.’ [16 p.1]. Health promotion also strives to reduce health inequalities [16]. Health literacy is another important concept in health promotion work: it includes the achievement of a level of knowledge, personal skills and ability for action in improving one’s health by changing one’s lifestyle and living conditions. It has been observed that people with low SES often have limited health literacy. Therefore, it is important for healthcare professionals to consider health literacy in their health-promoting work, especially when targeting people in low SES groups [17, 18].

Antonovsky’s salutogenic model of health is one basis for health promotion [19].

Salutogenesis means the origin of health [20]. In the salutogenic model, health is described as a continuum from a dis-ease pole of health to an ease pole. People’s resources are mobilised to deal with life events in the direction of the ease pole on the health continuum. The position on the continuum is not static but rather changes over time [21]. When stressors – i.e. something that causes a state of tension – appear in life, a person’s resources and ways of viewing the world around them will affect their ability to deal with the situation. The stressors will also affect their position on the health continuum with a focus on salutary factors [19, 21]. Antonovsky defined autogenesis as sense of coherence (SOC) [22]. SOC shows people’s view of life and their inner trust, which can improve their ability to detect and use the resources they have at their disposal [21]. A combined focus on stressors and resources might enable higher SOC, health and well-being [23].

SOC comprises three dimensions, the first of which is *comprehensibility.* This dimension has a cognitive perspective, meaning that the problem – or what happens internally or externally – is understood and clear. It also means that the person has the resources to make sense of the situation, even if this is challenging [20, 22]. With a clear understanding of problems or stressors, it is easier to deal with future stressors in life [23]. With high comprehensibility, people can figure out their own contexts and places where their role in different parts of society is essential, such as a workplace [24]. Comprehensibility could especially impact health behaviours that require people to have an active commitment to the activity, such as PA [25].

The second dimension is *manageability.* This dimension has a behavioural perspective whereby people use their resources to successfully cope with the situations or stressors at hand [20, 22]. The manageability dimension was created with inspiration from mastery and locus of control [23]. Some of the resources connected to manageability can be formal, such as healthcare professionals in public and private organisations, whereas others can be informal, such as people’s own resources or family, friends, colleagues or other significant persons [24, 26].

The third dimension, *meaningfulness*, has a motivational perspective whereby people find motivation to, a wish to, and a belief in their ability to cope with the situation. With a high degree of meaningfulness, people can feel that life is worth living even when sad things happen. In the meaningful dimension, people accept the challenge to seek meaning and overcome situations positively [20, 22]. With a high degree of this dimension, a person finds it meaningful to be able to solve different situations and has a desire for, is motivated for, and is willing to invest energy in solving the problem or to deal with the stressors that potentially cause distress [24]. According to Antonovsky, meaningfulness also leads people to ‘seek to order the world and to transform resources from potential to actuality’ [26 p.79].

General resistant resources (GRRs) are cornerstones in a strong SOC. Examples of GRRs can be material, such as having money, or immaterial, such as social support, coping strategies, identity, meaning of life such as religion or philosophy, the expression of being confident, a preventive orientation concerning health, mindset, knowledge and culture stability [23, 27]. The absence of a GRR can become a stressor [22]. This could involve, for instance, the absence of money. The absence of resources is called a general resistance deficit (GRD) [23]. Antonovsky combined the concepts of GRR and GRD to create one concept on a continuum (GRRs-RDs). A higher level on the continuum stands for viewing GRRs-RDs as GRR, while a lower level stands for viewing GRRs-RDs more as GRDs. GRRs-RDs can impact people’s SOC level, and their SOC level can influence them to find and use GRRs in dealing with tension [27]. Antonovsky also talked about specific resistance resources (SRRs), which refers to context-bound resources available in specific circumstances, such as telephone helplines; he also talked about the absence of SRRs as a specific resistance deficit (SRD) [23]. Economic difficulties are found more at the GRD pole on the GRR-RD continuum, while PA is found at the GRR pole [20]. As described above, people with lower SES, for instance having a strained economic situation, tend to engage less in PA. Still, there are people who achieve the WHO recommendations for PA despite adverse living conditions such as economic difficulties. There is a need for more knowledge regarding what may help people to be physically active despite adverse living conditions.

This study aims to explore how people, despite self-reported economic difficulties, comprehend, manage and find it meaningful to achieve the level of physical activity recommended by WHO.

## Method

### Design

In this study, a qualitative approach with individual semi-structured interviews was used, in combination with a structured questionnaire about sense of coherence.

### Setting

The study was conducted in southeast Sweden. In the regular work with health promotion at primary healthcare centres and family centres, inhabitants aged 40, 50, 60 or 70 years, as well as first-time parents, are invited to a targeted health dialogue. The Swedish concept of targeted population-based health dialogues is a health-promotive method whereby healthcare professionals, in dialogue with an individual, identify resources and risk factors. These dialogues support individuals in improving their lifestyle habits and reducing their risk factors for CVDs, in order to reach the ease pole on the health continuum [28–30]. If needed and desired, various follow-up visits can be conducted. When people comes to a health dialogue, they meet healthcare professionals such as district nurses, nurses, physiotherapists and occupational therapists trained in the health dialogue method, health promotion and motivational interviewing [28]. In the health dialogue a graphic pedagogical tool called the Health Curve is used, primarily focusing on lifestyle habits such as physical activity, food habits and the use of alcohol and tobacco, along with the individuals’ life situation and mental health, as reflected in their answers on the questionnaire. The questionnaire, described elsewhere [31], contains a question about experienced economic difficulties with the answers *yes*, *no* or *partly*. A physical activity score is calculated by means of a PA interview form. This calculation has been described elsewhere [32, 33]. To recruit participants to this study, people attending a targeted health dialogue at a primary healthcare centre or family centre were invited to participate.

### Inclusion criteria

To be included in this study, the following three inclusion criteria had to be met: (1) reporting having or partially having economic difficulties; (2) achieving a PA score of at least 750 points per week on the PA interview form during the health dialogue, corresponding to the lower limit of the WHO guidelines of 150 min per week; and (3) speaking Swedish well enough to participate in an interview.

The healthcare professionals were instructed to invite those who met the inclusion criteria to the present study.

### Data collection

For this study, we developed a semi-structured interview guide with questions about the participants’ experiences of PA in terms of motivation, support and perceived obstacles, as well as their experiences during and after PA (Supplemental material 1). The participants also responded to the short version of the sense of coherence questionnaire with 13 questions (SOC-13), developed by Aaron Antonovsky (with permission from the Avishai Antonovsky Society for Theory and Research for Salutogenesis https://stars-society.org/). Their statements and responses to the questions are rated on a semantic, unipolar verbal scale from 1 to 7 points, which are converted to SOC score points. The SOC-13 score ranges from 13 to 91 points, with 13 reflecting weak SOC and 91 strong SOC [24]. All interviews were conducted by the first author (LMJ) over the telephone. The SOC scale has previously been used in telephone interviews with reliable outcome [34]. The interviews lasted an average of 37.6 min, ranging from 29.6 to 48.5 min, and were transcribed verbatim.

### Participants

In this study, 17 persons (six men and eleven women) meeting the inclusion criteria agreed to participate and fulfilled the interview; three persons agreed to participate but then withdrew their participation before the interview was conducted. The participants were recruited from a county in south of Sweden with 368 856 inhabitants. The participants came from communities or cities with populations ranging from 5000 to 146 000 inhabitants, or their surrounding areas [35, 36]. Their average age was 45 years (SD 9 years), ranging between 28 and 60 years. They had an average of 2502 PA points, ranging from 810 to 5220 points. The participants’ SOC score from the SOC-13 questionnaire was ranging between 42 and 78 points.

### Data analysis

**Fig. 1:**
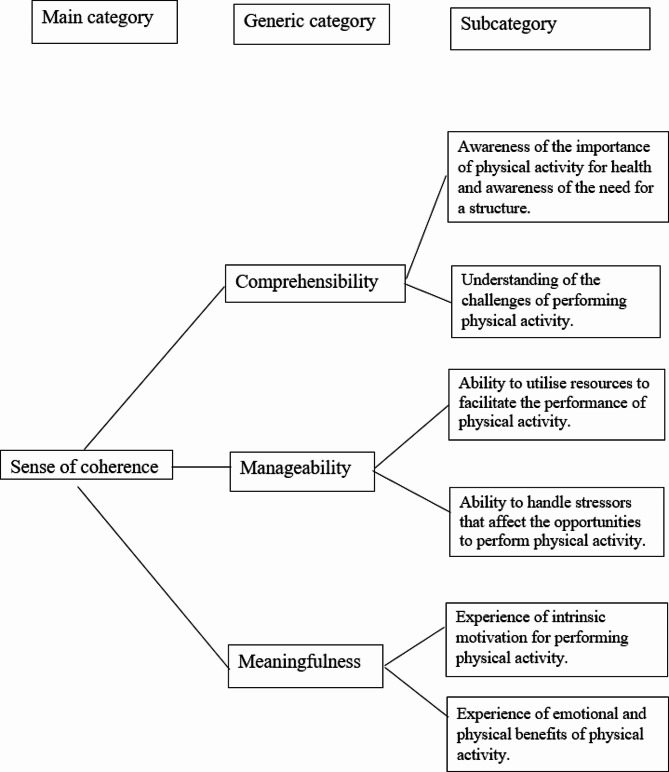
Overview of the categories from the analysis. Participants’ descriptions of their experiences of PA, analysed through comprehensibility, manageability and meaningfulness with their subcategories

Deductive qualitative content analysis according to Elo and Kyngäs was applied [37, 38]. The analysis matrix was constructed based on the three dimensions of SOC – *comprehensibility*, *manageability* and *meaningfulness* – which constituted the generic categories in the deductive analysis [37]. First, all transcribed interviews were read several times to obtain a sense of the content. During this process, notes and headings were written in the text with the generic categories in the analysis matrix in mind. The participants’ descriptions of their experiences of PA were identified and labelled with a code, and each code was placed in the corresponding generic category in the matrix. In the next step, the codes were grouped into subcategories in an ongoing inductive process until consensus was established, in order to enhance credibility [37, 39]. We also followed the Elo and Kyngäs checklist for content analysis to establish trustworthiness [39]. An overview of the categories is presented in Fig. 1. Two authors performed the analysis (LMJ and MG) and all authors read, commented on and discussed the analysis (LMJ, HL, EIF, MG).

The range of the SOC-13 is presented as background information.

### Ethical issues

The study received ethical approval from the Ethical Review Authority in Sweden, 2020 − 01249, with additional approvals #2021 − 00942, #2022-06806-02 and #2022-07198-0. Participation in the study was voluntary, and the participants gave written informed consent to participate. The participants had the opportunity to withdraw from the study at any time. The study was conducted in accordance with the Declaration of Helsinki.

## Results

The findings from the analyses are presented through the three categories of sense of coherence – comprehensibility, manageability and meaningfulness – with subcategories (Fig. 1).

### Sense of coherence

The findings indicate that the sense of coherence shows the participants’ capacity to cope with stressful situations in order to achieve the recommended levels of PA. The findings also reflect that the participants’ awareness of the importance of PA as an essential resource (GRR) is a prerequisite for being physically active. This awareness helps the participants to structure a plan for their PA and, together with their inner trust, identify their resources and the benefits of PA. The findings show how the participants – despite self-reported economic difficulties and other challenges and stressors – use their plans, resources, perceived benefits, and intrinsic motivation to achieve the recommended PA levels. The participants start from an understanding of the need for PA through a stage in which they use their resources to put their plan into practice and, with their intrinsic motivation also get the benefits of PA.

#### Comprehensibility

An awareness of the importance of PA and an understanding of the need for a structure are described as a basis for performing PA. Despite the participants’ knowledge and understanding of the importance of PA and their willingness to perform it, there are GRRs-RDs-stressors that might decrease their performed PA. The stressors that the participants mentioned were both external and internal.

#### Awareness of the importance of physical activity for health and awareness of the need for a structure

The participants described that they had knowledge and understanding of national and international guidelines regarding PA. They knew that PA that increases their heart rate is essential for preventing CVD and improving many other health aspects, such as sleeping well. It also emerged that the participants felt it was important that PA was performed in a healthy way, and that it was essential to adjust the PA to the age of the participant. The need for PA was also described as a habit from childhood, initiated by a significant other:

*In my home country, my dad always told me to go for a walk and to not sit by myself and look at the screen. It’s better if you go out. So almost every day, I had to go out with my dad for half an hour. It taught me that you have to walk and walk. If you sit at home, it’ll be a disaster. It’s better for the body to move.* Participant 16.

The participants described that PA might be good for them, and might help them to get a better structure for handling various problems in life. Thus, the plan for PA would help them to be better prepared to handle the world around them. Their plans dealt with different things, such as psychological problems and physical pain, becoming or staying healthy, and being better prepared to handle situations or illnesses that are to come:

*Of course I have this fibromyalgia, and the osteoarthritis is probably the worst. I have to keep myself alert and brisk and move, because otherwise my joints start to creak. I don’t want to have to take medicine and things like that. Walking is like my medicine.* Participant 4.

Participants pointed out the importance of PA despite a lack of time in a new situation, such as having a child or getting divorced. To deal with the new situation, the plan for PA had to be changed in relation to the way, place or time of the day or week that it could be performed. Knowledge about continuing to perform PA to stay healthy was essential. The participants also comprehended the consequences of different kinds of physical injuries and impairments, and understood that they had to handle these situations by changing their former PA plan.

#### Understanding of the challenges of performing physical activity

To make up plans for performing PA, the participants described that they had to understand their different challenges and stressors that may affect their performance of it. Time aspects emerged as an external stressor to performing PA: participants pointed out a lack of time as a factor resulting in less PA than intended, and prioritising their children or having to travel a long distance to work could take time from PA. Another external factor the participants mentioned was that a loss of their social network might negatively affect PA, especially when they had small children. The participants also mentioned the economy as a stressor that may affect the performance of PA. The economy might also affect what kind of activity they could afford, as well as their overall opportunity to perform PA due to their working situation. Other external stressors the participants mentioned were short hours of daylight during winter and bad weather restricting outdoor PA performance:

*Sometimes I wish I could go down a bit in time right now when I’m 60, but since I’m alone I have to work full time, which is taking a toll on me. Sometimes I feel like I could have reduced my working time if I’d been able to afford it, because I think I would have had more energy. Now I’ve had a week of vacation and I’ve been able to exercise whenever I wanted, but you realize the limit when you work like you do, that you can only do it in the evening.* Participant 8.

A typical internal stressor mentioned by the participants involved different kinds of physical injuries and impairments. Some of these problems decreased the opportunities to perform PA when it came to the way and the amount they had previously done. Tiredness, primarily due to a disease, was a factor that decreased and changed the opportunities to perform PA. Another internal stressor the participants mentioned was that they sometimes had to fight an inner resistance to perform PA and instead lay down and rested on the couch. From a psychological perspective, participants also stressed that the season could be important, with winter having a negative impact on their energy and mood to perform PA. Another stressor that emerged involved personal problems with performing PA together with other people.

#### Manageability

It emerged that the participants utilised their resources to facilitate the performance of PA. The GRRs on the GRR-RD continuum showed to be both formal and informal. The knowledge and understanding of the need for PA were adapted into the participants’ habits and ordinary activities. The analysis shows that the participants’ strategy for putting their PA plan into practice was based on their confidence in their own ability to perform PA.

#### Ability to utilise resources to facilitate the performance of physical activity

Formal resources for performing PA that emerged included getting help through the primary healthcare centre from physiotherapists, doctors or other personnel who advised the participants about PA and how to perform it in their situation. Examples mentioned included a training programme with help from healthcare professionals, or joint protection pads to use when performing PA in order to reduce the risk of injury or pain. It could also be the case that a workplace provided support for PA. A gym that offered a lower price for certain groups, for example those 60 years or older, was also mentioned as a form of support.

The informal resources participants mentioned included feeling support from their partner or having decided to perform PA together with their partner. Another facilitating example was performing PA in their own way with their own chosen activities. Furthermore, it emerged that it could be easier to perform PA if that time would otherwise have been spent just passively waiting for something or someone. One example the participants mentioned was using the time to perform PA themselves while their children had their own activity, instead of just sitting and waiting. Parking their car at some distance from their intended destination was another helpful behaviour. A flashlight was helpful in overcoming the lack of daylight in the wintertime. The participants also described how support from others in different kinds of group activities could facilitate their PA performance:

*Because alone it can easily be like, no, I’ll do it another day. But here you have someone, like, you can’t leave the others in the lurch; oh, then you just get going*. Participant 7.

Having a dog that needed to go out for a walk was also mentioned as being helpful for being physically active.

#### Ability to handle stressors that affect the opportunities to perform physical activity

The participants said it was helpful when they used their resources to make plans for PA in practice and let it become a habit in their lives. It was easier if it did not always have to be the same activity or duration; it was just the habit of performing PA itself that was important. Different behavioural strategies were also important, such as bringing one’s training clothes to work and performing the activity before returning home. Another helpful strategy involved booking gym workouts beforehand so that one felt forced to show up. Participants also pointed out the importance of accepting different unexpected situations and being open to changing their behaviour according to the situation at hand. One example involved reducing the time spent on each PA session, and instead performing shorter sessions for more days each week. A divorce situation in which the participant’s children lived with them every second week might affect their possibilities to perform PA; this could be solved by more PA performance during the weeks without the children and less during the other weeks. An openness to new activities during the weeks without the children due to having more time for PA was also described. It was also said to be helpful to have the ability to change focus from one activity to another. The participants described themselves as determined people with confidence in their PA performance. According to them, it was only they themselves who could get it done:

*I think it’s fun to exercise anyway – things where you use your body. I have quite a heavy workload during the day, but it’s possible anyway because I always challenge myself. I never give up. That’s how I think about it.* Participant 12.

Another example was that a habit of being physically active generated more PA.

#### Meaningfulness

The participants’ intrinsic motivation and belief in their ability to cope with the PA-related situations and overcome the challenges and stressors in their lives in a positive perspective emerged as essential to them. They associated many things, such as calm and harmony, to PA and it made life worth living. They needed to get positive benefits from the energy they invested in performing PA in order to feel like it was meaningful; these benefits could be either emotional or psychological, as well as physical.

#### Experience of intrinsic motivation for performing physical activity

The participants expressed that they had an inner drive that motivated them to perform PA. One motivational factor was their positive attitude towards their own will to perform PA, even though they were concerned about being unable to cope or having time for everything. Another motivational factor that emerged involved overcoming pain and other physical problems and disease symptoms by performing PA. Furthermore, the participants performed PA based on a motivation to maintain their strength, and a wish to have energy when they got older as well as to be better able to overcome possible future diseases or accidents. Other motivating factors they described included being attractive, losing weight and having a body with less fat and more muscle. Another motivation could involve being able to eat what they wanted, compensating for unhealthy food habits through PA. Participants also mentioned as an example that the fellowship they experienced in a sports club, where they were confirmed as a person and had the chance to help others with PA, motivated their PA performance. Using PA to overcome situations instead of taking medication was another motivational factor.

Participants described that a solid motivation for performing PA involved being there for their children or grandchildren in the future, and being active with them even in the present time. Their children and grandchildren motivated them to be physically active, with the perspective of having better health and a longer life:

*A little more philosophically, when we found out we were having a baby, I thought that being physically active is very strongly linked to longevity, how long; I mean, my chances and opportunities to live a long time and to be there for my child. So, if you look at the bigger picture. I think life is an extra motivation, that there’s a common thread through everything we do. If I want to be here for my child, then, of course, I want to influence all the possibilities to be here as much as possible, so that brings in being physically active.* Participant 1.

The benefits of PA included good sleep and feeling good mentally. Other examples that motivated PA included the opportunity to spend time with their pets, such as dogs, or to simply spend time in nature. The participants said that they became motivated through their favourable experiences of PA, and that they felt good and had fun performing PA.

#### Experience of emotional and physical benefits of physical activity

The participants described feeling emotionally good in their whole body after performing PA. Another benefit was that the atmosphere at home became positive. Other effects included an emotional feeling of joy and mental strength. PA also provided the benefits of feeling more harmony and satisfaction. It resulted in more vitality, creativity and energy for everyday life. Another benefit that emerged was that restlessness was transformed into calmness, but still with positive energy:

*I get more energy at the gym, or when I’ve done some physical activity; I feel very alert.* Participant 8.

It emerged that performing PA could have the psychological benefit of strengthening one’s self-esteem. Another positive psychological benefit was that it was easier to plan one’s day when performing PA and to solve any problems that arose. The participants described that after performing PA they became calmer, less stressed, and less angry and sad. They also described that performing PA prevented negative thoughts and made it easier to keep their mood up. Furthermore, they described that they became more congenial to themselves and others. PA was also described as being good for the brain:

*I feel good when I’ve exercised. I feel comfortable, with no stress and no bad thoughts.* Participant 16.

The participants described that PA made them feel physically better in their bodies. It also emerged that they became tired in a positive way, and slept better and more deeply after PA. Other positive benefits of PA they described included improved appetite, body posture and balance. It also emerged that, despite muscle soreness after performing PA, they had a good feeling in their bodies and experienced less pain and stiffness.

## Discussion

The findings of this study elucidate how sense of coherence may explain how people, despite economic difficulties, achieve the WHO recommendations for physical activity.

The SOC-13 score of indicates a relatively strong SOC The possibility to achieve the recommendations for PA can be described as a process. This process, which emerged from the analysis, starts with an awareness of the need for PA through a stage in which the participants use their resources to set their PA plan in practice. Their PA performance is also facilitated through their intrinsic motivation and the benefits of PA. This process, which shows how the dimensions of SOC interact with each other in a direction towards the ease pole on the health continuum, may facilitate for participants to achieve the recommendations for PA despite challenges in their lives. The analysis aligns with the unique SOC concept, with the trilateral combination and interaction between the cognitive, behavioural and motivational dimensions of SOC [40].

In one study, it has been shown that persons with a weak SOC also report low PA [41]. Another study showed that PA was associated with a stronger SOC as well as good psychological and social health [42]. The results in both these studies are in line with the present study.

The first generic category, comprehensibility, showed the importance of knowledge of the health benefits of PA and an awareness in applying this knowledge in a plan for PA. The knowledge became the foundation in a process for the participants to achieve the PA recommendations. They showed cognitive skills in applying their knowledge in a PA plan, which helped them to structure different life problems; this is in line with other studies [22, 23]. Still other studies, taking health literacy into consideration, have shown that health-promoting work in which the knowledge and understanding of the importance of PA – which can be appraised and applied in people’s lives– is essential to achieve different health goals such as PA recommendations [17, 18, 43]. Another study among middle-aged women showed the importance of knowing how to perform PA; they did not perform PA because they did not know how to do it [44]. Comprehensibility might also affect health behaviours requiring personal commitment [25]. Medical conditions such as pain and various diagnoses were mentioned as challenges and were considered in the PA plan so that the activities fit the medical condition, as has been shown in other studies as well [45]. The findings in the present study showed that economic difficulties were seen as a challenge in regard to performing PA, for instance what kind of activity one could afford and the possibility to perform PA with respect to one’s work situation. It has also been shown that economic difficulties in the GRD direction on the GRR-RDs continuum can be overcome [23]. Another previous study showed that persons without economic problems spent more time performing PA than those with economic difficulties did [15]. Other challenges among participants were a lack of time and social support for PA, which has also been seen in other studies [44, 45]. Participants in the current study described that PA as a habit from their childhood could be part of their awareness of it and facilitate their PA plan. This was also confirmed in another study [46].

In the second generic category, the behavioural category of manageability, the participants described their ability to utilise their resources and how they handle stressors that might negatively affect their achievement of the PA recommendations, in accordance with Antonovsky [22]. One of the formal resources the participants mentioned was support from healthcare professionals, aligning with an earlier study from Norway [47]. Other resources the participants reported involved performing the activity in a group or with their partner, and doing activities they had chosen themselves. These factors were also found by Barker and Eickmeyer [45, 48]. The participants in the current study described being out in nature with their dog as a resource for performing PA. In a systematic review it was also found that taking the dog out facilitated physical activity for middle-aged persons [49]. The participants in the current study described themselves as determined persons with self-efficacy in performing PA. This aligns with a systematic review that showed the importance of self-efficacy in performing PA [50].

The third generic category is the motivational category of meaningfulness, in which participants in the current study described their intrinsic motivation and perceived benefits of PA. They also described their positive attitude towards performing PA, aligning with another study showing that intrinsic motivation is essential to performing PA [51]. Participants in the current study also found it meaningful to perform PA due to its mental and emotional benefits. These benefits are also described elsewhere [15, 50, 52]. Other benefits of PA were higher satisfaction with their body, such as losing or maintaining weight, having less pain, maintaining health, having more strength, better sleep, and to overcome disease-related problems, which also has been seen in other studies [44–46]. Participants in the current study also described more energy and a feeling of vitality as essential benefits of PA. Other studies have also shown the association of PA with energy and vitality [7, 46].

A summary of the main findings and supporting references is provided in supplemental material 2.

### Strengths and limitations

A strength of this study is that the data from the 17 interviews were rich. For trustworthiness in the organising phase of the content analysis, rich data facilitates the replication in the categories [39]. During the preparing phase, organising phase and reporting phase, we also followed the Elo and Kyngäs checklist for content analysis to establish trustworthiness [39]. Other strengths were that the participants came from both rural and urban areas, and were of different ages. Furthermore, the participants referred to different diseases, impairments and pain. This may indicate that they represent people that have harder to perform PA than the general population. Despite this, they reported a high level of PA.

The inclusion criteria regarding PA and economic difficulties were based on the participants’ self-reports. While self-reported data may be imprecise, the PA interview form used in health dialogues is validated, and no systematic over- or underestimation of PA has been observed [33]. In another study, it has been found that self-reported economic difficulties may differ from objective measures of economic situation, which might have affected the inclusion of participants in our study. Regarding health, the same study showed a stronger predictive validity for self-reported economic difficulties than for objective measures of economic situation [53].

## Conclusion

The analysis in this study shows a pattern of a possible process of how participants with a relatively strong SOC may comprehend, manage and find it meaningful to achieve the recommended PA levels despite economic difficulties. In this process, the participants were aware of the importance of being physically active and of the need for a structured plan for achieving the recommended PA goals. They also understood the challenges of performing PA. They described how they followed and adjusted their plans, and how they utilised their resources in order to handle stressors that may aggravate PA. They described their intrinsic motivation for PA and experienced benefits from it as essential to find it meaningful to be physically active. The process and examples from our study might be helpful in health-promoting work, such as targeted health dialogues with persons who do not achieve the PA recommendations in primary health care.

### Electronic supplementary material

Below is the link to the electronic supplementary material.

Supplementary Material 1

Supplementary Material 2

## Data Availability

The dataset generated and/or analysed during the current study are not publicly available due to privacy and ethical restriction, but are available from corresponding author on reasonable request.

## Abbreviations

CVD: Cardiovascular diseases
GRD: General resistance deficit
GRR: General resistant resources
GRRs-RD: A combined the concepts of GRR and GRD on a continuum
LTPA: Leisure time physical activity
NCD: Non-communicable diseases
PA: Physical activity
SES: Socioeconomic status
SOC: Sense of coherence
WHO: World Health Organisation
