# Supplementary material for: Exploring how people achieve recommended levels of physical activity, despite self-reported economic difficulties: a sense of coherence perspective
Source: BMC Prim Care. 2024 Apr 4;25:105. doi: 10.1186/s12875-024-02354-z (PMC10993487; doi:10.1186/s12875-024-02354-z)
Supplement: Supplementary file 1 — Supplementary Material 1 [file 12875_2024_2354_MOESM1_ESM.docx]

**Interview Guide**

Supplemental material1

How did you experience the health dialogue?

What did you talk about the most?

Can you describe the part of the health dialogue that was about physical activity?

How much did you talk about physical activity?

Did you get the help you wanted, based on the dialogue?

Have you thought whether you would like to change or have recently made a change of your physical activity?

- If so, what do you like to change?

- How confident are you that you could make that change?

How would you describe how you feel when you have been physically active?

What motivates you to be physically active?

Is there anything you would find as helpful to perform physical activity?

Is there anything you would experience as an obstacle to performing physical activity?

How would you describe your health in general?

**Follow up questions**

Do you want to tell me more about that?

Would you like to elaborate a bit more?

Can you give me an example?

Can you describe what you mean?
