# Supplementary material for: Exploring how people achieve recommended levels of physical activity, despite self-reported economic difficulties: a sense of coherence perspective
Source: BMC Prim Care. 2024 Apr 4;25:105. doi: 10.1186/s12875-024-02354-z (PMC10993487; doi:10.1186/s12875-024-02354-z)
Supplement: Supplementary file 2 — Supplementary Material 2 [file 12875_2024_2354_MOESM2_ESM.docx]

Supplemental material 2

Supplemental table 1. A summary of the findings with supporting references.

| Findings | Supporting references |
| --- | --- |
| A process emerged from the analysis, which starts with an awareness of the need for PA through a stage in which the participants use their resources to set their PA plan in practice. Their PA performance is also facilitated through their intrinsic motivation and the benefits of PA. This process, which shows how the dimensions of SOC interact with each other in a direction towards the ease pole on the health continuum, may facilitate for participants to achieve the recommendations for PA despite challenges in their lives. | Antonovsky A: **The salutogenic model as a theory to guide health promotion**. 1996, **11**(1):11-18. |
| Persons with a weak SOC also report low PA. | Thomas K, Nilsson E, Festin K, Henriksson P, Lowén M, Löf M, Kristenson M: **Associations of Psychosocial Factors with Multiple Health Behaviors: A Population-Based Study of Middle-Aged Men and Women**. *Int J Environ Res Public Health* 2020, **17**(4). |
| PA was associated with a stronger SOC as well as good psychological and social health. | Read S, Aunola K, Feldt T, Leinonen R, Ruoppila I: **The relationship between generalized resistance resources, sense of coherence, and health among Finnish people aged 65-69**. *European psychologist* 2005, **10**(3):244-253. |
| The importance of knowledge of the health benefits of PA and an awareness in applying this knowledge in a plan for PA. The knowledge became the foundation in a process for the participants to achieve the PA recommendations. They showed cognitive skills in applying their knowledge in a PA plan, which helped them to structure different life problems | Forbech Vinje H, Langeland E., Bull T.: **Aaron Antonovsky's Development of Salutogeneses, 1979-1994** In: *The Handbook of Salutogenisis Second edition.* Edited by Mittelmark MB, Bauer GF, Vaandreger L, Pelikan JM, Sagy S, Eriksson M, Lindström B, Meier Magistretti C, Barry MM; 2022.  Eriksson M, Contu P.: **The Sence of Coherence: Mesurement Issues**. In: *The Handbook of Salutogenesis Second edition.* Edited by Mittelmark MB, Bauer GF, Vaandreger L, Pelikan JM, Sagy S, Eriksson M, Lindström B, Meier Magistretti C, Barry MM; 2022. |
| Health literacy, have shown that health-promoting work in which the knowledge and understanding of the importance of PA – which can be appraised and applied in people’s lives– is essential to achieving different health goals such as PA recommendations. | Phillips A:  **Effectiveapproaches to health promotion in nursing practice**. *Nursing standard (Royal College of Nursing (Great Britain) : 1987)* 2019, **34**(4):43-50.  Nutbeam D: **Health literacy as a public health goal: a challenge for contemporary health education and communication** **strategies into the 21st century.**  *Health promotion international* 2000, **15**(3):259-267    Ericson H, Quennerstedt M, Skoog T, Johansson M: **Health resources, ageing and physical activity: a study of physically active women aged 69-75 years**. *Qualitative Research in Sport, Exercise and Health* 2018, **10**(2):206-222. |
| Importance of knowing how to perform PA. | Crooke R, Haseler C, Haseler T, Collins J, Crockett A: **Physical activity and moving more for health**. *The journal of the Royal College of Physicians of Edinburgh* 2020, **50**(2):173-180. |
| Comprehensibility affect health behaviours requiring personal commitment. | Adorni R, Zanatta F, D'Addario M, Atella F, Costantino E, Iaderosa C, Petarle G, Steca P: **Health-Related Lifestyle Profiles in Healthy Adults: Associations with Sociodemographic Indicators, Dispositional Optimism, and Sense of Coherence**. *Nutrients* 2021, **13**(11):3778. |
| Medical conditions such as pain and various diagnoses were mentioned as challenges and were considered in the PA plan so that the activities fit the medical condition. | Barker K, Eickmeyer S: **Therapeutic Exercise**. *The Medical clinics of North America* 2020, **104**(2):189-198. |
| Economic difficulties were seen as a challenge in regard to performing PA, for instance what kind of activity one could afford and the possibility to perform PA with respect to one’s work situation. It has also been shown that economic difficulties in the GRD direction on the GRR-RDs continuum can be overcome. | Forbech Vinje H, Langeland E., Bull T.: **Aaron Antonovsky's Development of Salutogeneses, 1979-1994** In: *The Handbook of Salutogenisis Second edition.* Edited by Mittelmark MB, Bauer GF, Vaandreger L, Pelikan JM, Sagy S, Eriksson M, Lindström B, Meier Magistretti C, Barry MM; 2022.  Johansson LM, Lingfors H, Golsater M, Kristenson M, Fransson EI: **Can physical activity compensate for low socioeconomic status with regard to poor self-rated health and low quality-of-life?** *Health Qual Life Outcomes* 2019, **17**(1):33 |
| Challenges to perform PA among participants were a lack of time and social support for PA. | Crooke R, Haseler C, Haseler T, Collins J, Crockett A: **Physical activity and moving more for health**. *The journal of the Royal College of Physicians of Edinburgh* 2020, **50**(2):173-180.    Barker K, Eickmeyer S: **Therapeutic Exercise**. *The Medical clinics of North America* 2020, **104**(2):189-198. |
| PA as a habit from their childhood could be part of their awareness of it and facilitate their PA plan. | Ericson H, Quennerstedt M, Geidne S: **Physical activity as a health resource: a cross-sectional survey applying a salutogenic approach to what older adults consider meaningful in organised physical activity initiatives**. *Health Psychology and Behavioral Medicine* 2021, **9**(1):858-874. |
| The participant ability to utilise their resources to handle stressors that might negatively affect their achievement of the PA recommendations. | Antonovsky A: **Unraveling the mystery of health** San Francisco, Calif.: Jossey-Bass; 1987. |
| A formal resource the participants mentioned was support from healthcare professionals. | Falskog F, Landsem AM, Meland E, Bjorvatn B, Hjelle OP, Mildestvedt T: **Patients want their doctors' help to increase physical activity: a cross sectional study in general practice**. *Scandinavian journal of primary health care* 2021, **39**(2):131-138. |
| Performing the activity in a group or with their partner, and doing activities they had chosen themselves were seen as resources. | Barker K, Eickmeyer S: **Therapeutic Exercise**. *The Medical clinics of North America* 2020, **104**(2):189-198.  Ericson H, Quennerstedt M, Skoog T, Johansson M: **Health resources, ageing and physical activity: a study of physically active women aged 69-75 years**. *Qualitative Research in Sport, Exercise and Health* 2018, **10**(2):206-222 |
| A resource to perform PA is to be out in the nature with their dogs. | Spiteri K, Broom DR, Bekhet AH, de Caro JX, Laventure B, Grafton K: **Barriers and Motivators of Physical Activity Participation in Middle-aged and Older-adults - A Systematic Review**. *Journal of aging and physical activity* 2019:1-80. |
| The participants described themselves as determined persons with self-efficacy in performing PA. | Medrano-Ureña MDR, Ortega-Ruiz R, Benítez-Sillero JD: **Physical Fitness, Exercise Self-Efficacy, and Quality of Life in Adulthood: A Systematic Review**. *Int J Environ Res Public Health* 2020, **17**(17). |
| Participants described their intrinsic motivation and perceived benefits of PA. They also described their positive attitude towards performing PA. | Molanorouzi K, Khoo S, Morris T: **Motives for adult participation in physical activity: type of activity, age, and gender**. *BMC public health* 2015, **15**:66. |
| Participants found it meaningful to perform PA due to its mental and emotional benefits. | Johansson LM, Lingfors H, Golsater M, Kristenson M, Fransson EI: **Can physical activity compensate for low socioeconomic status with regard to poor self-rated health and low quality-of-life?** *Health Qual Life Outcomes* 2019, **17**(1):33.  Medrano-Ureña MDR, Ortega-Ruiz R, Benítez-Sillero JD: **Physical Fitness, Exercise Self-Efficacy, and Quality of Life in Adulthood: A Systematic Review**. *Int J Environ Res Public Health* 2020, **17**(17).  Lee MN, Kim SD, Choi YS: **The Relationship between Physical Activity and Health-Related Quality of Life (HINT-Eight) in Middle-Aged Korean Women**. *Journal of environmental and public health* 2022, **2022**:4555547 |
| Satisfaction with appreciation of the body, such as losing or maintaining weight, having less pain, maintaining health, having more strength, better sleep, and to overcome disease-related problems were experienced as benefits of PA. | Crooke R, Haseler C, Haseler T, Collins J, Crockett A: **Physical activity and moving more for health**. *The journal of the Royal College of Physicians of Edinburgh* 2020, **50**(2):173-180.  Barker K, Eickmeyer S: **Therapeutic Exercise**. *The Medical clinics of North America* 2020, **104**(2):189-198.  Ericson H, Quennerstedt M, Geidne S: **Physical activity as a health resource: a cross-sectional survey applying a salutogenic approach to what older adults consider meaningful in organised physical activity initiatives**. *Health Psychology and Behavioral Medicine* 2021, **9**(1):858-874 |
| Participants described more energy and a feeling of vitality as essential benefits of PA. | Johansson LM, Lingfors H, Golsäter M, Kristenson M, Fransson EI: **Physical activity related to mastery and vitality in a Swedish adult population with economic difficulties**. *BMC public health* 2021, **21**(1):2193.  Ericson H, Quennerstedt M, Geidne S: **Physical activity as a health resource: a cross-sectional survey applying a salutogenic approach to what older adults consider meaningful in organised physical activity initiatives**. *Health Psychology and Behavioral Medicine* 2021, **9**(1):858-874. |
